# Yu Linzhu alleviates primary ovarian insufficiency in a rat model by improving proliferation and energy metabolism of granulosa cells through hif1α/cx43 pathway

**DOI:** 10.1186/s13048-024-01408-1

**Published:** 2024-04-26

**Authors:** Xin Ruan, Pengxu Wang, Maolin Wei, Qingqing Yang, Xiaoying Dong

**Affiliations:** Department of Traditional Chinese Medicine, Institute of Traditional Chinese Medicine, Capital Medicine University, Beijing, 100069 China

**Keywords:** Yu Linzhu (YLZ), Primary Ovarian Insufficiency (POI), Granulosa cells, HIF1α /CX43 Pathway, Follicular development

## Abstract

**Background:**

Yu Linzhu (YLZ) is a classical Chinese traditional formula, which has been used for more than 600 years to regulate menstruation to help pregnancy. However, the mechanism of modern scientific action of YLZ needs to be further studied.

**Methods:**

Thirty SD female rats were divided into three groups to prepare the blank serum and drug-containing serum, and then using UHPLC-QE-MS to identify the ingredients of YLZ and its drug-containing serum. Twenty-four SD female rats were divided into four groups, except the control group, 4-vinylcyclohexene dicycloxide (VCD) was intraperitoneally injected to establish a primary ovarian insufficiency (POI) model of all groups. Using vaginal smear to show that the estrous cycle of rats was disturbed after modeling, indicates that the POI model was successfully established. The ELISA test was used to measure the follicle-stimulating hormone (FSH), estradiol (E2), and anti-Mullerian hormone (AMH) levels in the serum of rats. HE stain was used to assess the morphology of ovarian tissue. The localization and relative expression levels of CX43 protein were detected by tissue immunofluorescence. Primary ovarian granulosa cells (GCs) were identified by cellular immunofluorescence. CCK8 was used to screen time and concentration of drug-containing serum and evaluate the proliferation effect of YLZ on VCD-induced GCs. ATP kit and Seahorse XFe24 were used to detect energy production and real-time glycolytic metabolism rate of GCs. mRNA and protein expression levels of HIF1α, CX43, PEK, LDH, HK1 were detected by RT-PCR and WB.

**Results:**

UHPLC-QE-MS found 1702 ingredients of YLZ and 80 constituents migrating to blood. YLZ reduced the FSH while increasing the AMH and E2 levels. In ovarian tissues, YLZ improved ovarian morphology, follicle development, and the relative expression of CX43. In vitro studies, we found that YLZ increased the proliferative activity of GCs, ATP levels, glycolytic metabolic rate, HIF1α, CX43, PEK, HK1, LDH mRNA, and protein levels.

**Conclusions:**

The study indicated that YLZ increased the proliferation and glycolytic energy metabolism of GCs to improve follicular development further alleviating ovarian function.

**Supplementary Information:**

The online version contains supplementary material available at 10.1186/s13048-024-01408-1.

## Introduction

Primary ovarian insufficiency (POI) is a disease in women that is defined as the loss or dysfunction of the follicles within the ovary associated with amenorrhea before the age of 40, resulting in low estrogen levels, rare amenorrhea or amenorrhea, and decreased fertility [1]. The incidence of POI is increasing year by year and tends to be younger. A recent meta-analysis shows that the incidence of POI is as high as 3.7% globally, and the natural pregnancy rate of POI patients is only 1.5–4.4%, which seriously affects the reproductive health of women in childbearing age [2]. At present, most scholars believe that the occurrence of POI is closely related to autoimmune, genetic, environmental factors, radiotherapy, and chemotherapy [1]. Among them, with the development of society, environmental factors have increasingly become a very crucial factor affecting the occurrence of POI. 4-vinylcyclohexene diepoxid (VCD) is a commonly used industrial chemical and environmental pollutant, widely found in rubber products, pesticides, spices, plasticizers, and other common daily goods, with strong reproductive toxicity [3, 4]. Flaws et al. reported as early as 1994 that VCD can target the destruction of oocytes in the ovaries of mature rats, two years later, he pointed out that small preantral follicles are more susceptible to VCD ovotoxicity, which may reduce follicle viability by affecting the viability of GCs, the companion of oocytes [5, 6]. Wei et al. recently found that VCD caused follicular atresia by influencing the apoptosis of GCs [7]. Previous studies have shown that VCD is an ideal and classical chemical agent for establishing POI models [8–10].

The key pathological link of POI is the developmental disorder from primordial follicles to antral follicles. The follicle's development at this stage mainly depends on the normal function of ovarian granulosa cells (GCs) and oocytes. Therefore, how to inhibit the excessive depletion of follicles and promote their normal development has important practical significance for improving the pregnancy rate of women. GCs are essential for the development of follicles, and their abnormal function is a key factor leading to follicle atresia [11]. During follicle development, GCs are divided into two anatomically and functionally distinct cell subtypes; parietal granulosa cells and cumulus granulosa cells [12]. Cumulus granulosa cells are in direct contact with oocytes and transmit amino acids, nucleotides, and glycolytic metabolic substrates to oocytes through gap junctions, and oocytes secrete some growth factors such as Bone morphogenetic protein 15 (BMP15) and Growth/differentiation factor 9 (GDF9) to GCs promoting their proliferation and differentiation in a complementary manner [13]. Since oocytes as the largest cells in mammals, consuming more energy for their development than somatic cells, and the lack of key glycolytic enzymes makes them critically dependent on glycolytic metabolic substrates given by GCs [14]. As a key target of glycolysis, hypoxia-inducible factor-1alpha (HIF1α) plays a crucial role in the pathway of regulating energy metabolism in GCs, and it promotes the occurrence of glycolytic biological processes by initiating downstream glycolysis-related targets [15]. Connexin 43 (Cx43) is a key protein mediating intercellular communication in follicles, and its absence directly inhibits the proliferation of GCs [16].

**Fig. 1:**
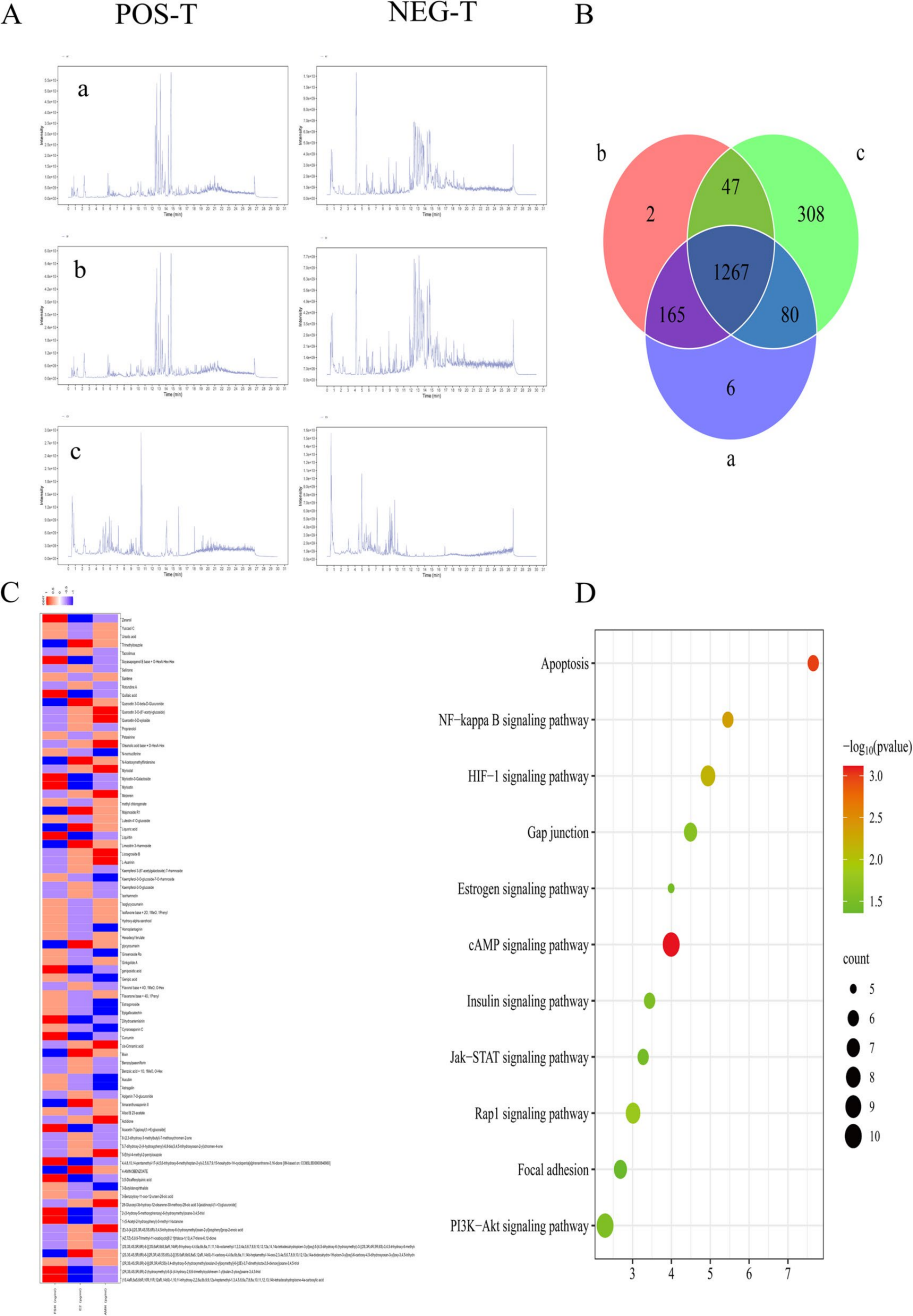
(A) Quality Control of YLZ and drug-containing serum by UHPLC-MS. (a) the chromatograms of drug-containing serum, (b) blank serum, (c) YLZ decocting solution in positive and negative ion mode; (B) veen map of the intersection ingredients; (C) heat map of correlation between enrolment ingredients (the intersection of a and c) and serum hormones; (D) bubble map of KEGG enrichment analysis

Although there are many drugs for the treatment of POI, the current mainstream method remains in the HRT (hormone replacement therapy) stage, for its hormone, optimal dose, duration, and many side-effects caused by the long-term application, such as the risk of breast cancer, endometrial cancer, and other diseases are uncontrollable, which increases the difficulty of the treatment of POI to a certain extent [17]. In addition, HRT can improve the systemic symptoms of POI to a certain extent, but cannot promote the normal development of follicles, which is also the reason why HRT cannot be the core therapy for the treatment of POI [17]. Traditional Chinese medicine has great potential and advantages in the prevention and treatment of POI due to its characteristics of overall regulation, multi-target, and multi-pathway [18]. Traditional Chinese medicine (TCM) has been more and more widely accepted worldwide, Yu Linzhu (YLZ) as a Chinese medicine formula for regulating menstruation and assisting pregnancy in the classics of TCM, derives from Complete Works of Zhang Jingyue (Ming Dynasty). The whole formula tonifying kidney essence and kidney qi, nourishes spleen and stomach to replenish qi and blood, promoting follicular development and then improving ovarian function by regulating the balance of essence and qi in the kidney (YLZ maintains the balance of energy relationship between oocytes and GCs in benign homeostasis). YLZ has been extensively used in clinical treatment of POI and basic research, clinical studies have shown that it can effectively regulate the level of sex hormones and reduce clinical symptoms in patients with POI with high safety, thus further improving ovarian function [19]. Basic studies have found that it can improve ovarian function in mice by improving oocyte mitochondrial function, ovarian oxidative stress, and ovarian microenvironment [20–22]. Previously, we have performed Kyoto Encyclopedia of Genes and Genomes (KEGG) pathway enrichment analysis about YLZ treatment of POI (Fig. 1D), and based on the research direction and content of our project, we selected the HIF1α/Cx43 signaling pathway for validation. We speculated that YLZ could improve POI status in rats by promoting energy metabolism and proliferation of GCs through the HIF1α/Cx43 signaling pathway.

This study aims to explore the therapeutic potential of YLZ, a traditional Chinese medicine formula, in alleviating POI. The primary objectives involve evaluating the incidence and severity of POI in a rat model induced by VCD, with a focus on assessing disrupted estrous cycles and altered levels of reproductive hormones. Additionally, the study seeks to investigate the effects of YLZ on critical aspects of ovarian function, including morphology, follicle development, and the expression of key proteins such as Cx43, pivotal for intercellular communication in follicles. Furthermore, the research aims to assess how YLZ influences the proliferation and energy metabolism of GCs, essential components for effective follicular development. Finally, the study aims to delve into the modulation of the HIF1α /Cx43 signaling pathway by YLZ, with the overarching goal of elucidating the molecular mechanisms underlying the therapeutic effects of this traditional Chinese medicine formula on POI.

## Materials and methods

### Materials and reagents

The following list of reagents was mainly utilized in this study: VCD (Sigma, USA); YLZ herbs (Beijing Tong Ren Tang, China); Dulbecco's Modified Eagle Medium (DMEM) (Gibco, USA); D-hanks solution (AQ, China); Fetal Bovine Serum (FBS) (Gibco, USA); follicle-stimulating hormone receptor (FSHR) (Bioss, China); Immunoglobulin G (IgG) (ZSZSGBBIO, China); 4% polyformaldehyde (KeyGEN Biotech, China); Estradiol (E2) (Raybio, USA); follicle-stimulating hormone (FSH), E2, Anti-mullerian hormone (AMH) enzyme-linked immunosorbent assay (ELISA) (Elabscience, China); Seahorse Xfe 24 kit (Alicelligent, China); Adenosine Triphosphate (ATP) test kit (Beyotime, China); Guanidine isothiocyanate (TRIzol) (Vazyme, China); RIPA Lysis Buffer (RIPA) (Beyotime, China); Polyvinylidene Fluoride (PVDF), Enhanced Chemiluminescence Reagent (ECL) (Millipore, USA); CX43 antibody (Servicebio, China); HIF1α antibody (Affinity, USA); Phospho-Thr981 antibody (PEK), Hexokinase1 antibody (HK1) (Proteintech, USA); Lactate Dehydrogenase antibody (LDH) (WANLEIBIO, China); Glyceraldehyde-3-phosphate dehydrogenase antibody (GAPDH) (CST, USA).

### Preparation of YLZ and drug-containing serum

YLZ herbs were weighed as the Table 1. Firstly, Lu Jiaoshuang (LJS) was boiled 30 min, ten times the amount of distilled water was added, followed by 30 min of soaking, 45 min of boiling, filtering, and second boil with 8 times the amount of distilled water, Ren Shen (RS) was boiled 1 h separately, filtering, the three solutions were finally mixed and the drug concentration of YLZ was 1.1 g/ml, finally, stored at 4℃after it cooled naturally. Mixing the estradiol valerate tablets and distilled water into suspension completely, and stored. After one week of adaptive feeding, thirty female Sprague Dawley (SD) rats (300 ± 50 g, 8–9 weeks old, purchased from Beijing WTLH, China, Experimental Animal Use License No. SCXK (Beijing) 2021–0011) Approval Number of Animal Ethics Committee of Capital Medical University: AEEI-2021–131) were randomly divided into 3 groups (n = 10/ group): normal serum group (given the same volume of distilled water as YLZ), YLZ serum group (10.08 g/kg, twice a day) and estradiol serum group (0.1008 mg/kg, twice a day). All drugs were administered intragastric for 5 days. The doses of YLZ and estradiol valerate tablets were converted from clinical human doses to rat doses and combined with our findings from earlier studies. One hour after the last gavage, the rats were anesthetized with isoflurane, and blood was collected from the rat's abdominal aorta and centrifuged at 4℃ and 3000 rpm for 15 min. The complement was inactivated (56℃, 30 min) and bacteria were removed through a 0.22 μm filtration membrane, and then stored at -80℃ for subsequent experiments.

**Table 1:** Composition of YLZ

CHN Pinyin	Latin name	CHN	Family	Amount (g)
Ren Shen (RS)	Panax ginseng C. A. Mey	人参	Araliaceae	6
Fu Ling (FL)	Poria cocos	茯苓	Polyporaceae	6
Bai Zhu (BZ)	Atractylodes macrocephala Koidz	白术	Asteraceae	6
Gan Cao (GC)	Radix Glycyrrhizae	甘草	Leguminosa	3
Dang Gui (DG)	Angelica Sinensis	当归	Umbelliferae	12
Shu Di (SD)	Rehmanniae preparata Radix	熟地	Scrophulariaceae	12
Chuan Xiong (CX)	Ligusticum sinense	川芎	Umbelliferae	3
Bai Shao (BS)	Cynanchum otophyllum Schneid	白芍	Asclepiadaceae	6
Tu Sizi (TSZ)	Cuscutae Semen	菟丝子	Convolvulus	12
Du Zhong (DZ)	Eucommia Ulmoides Oliv	杜仲	Eucommiaceae	6
Lu Jiaoshuang (LJS)	Cervus nippon Temminck	鹿角霜	Cervidae	6
Hua Jiao (HJ)	Zanthoxylum bungeanum Maxim	花椒	Rutaceae	6

### Ultra High Performance Liquid Chromatography-Q Exactive-Mass Spectrometer (UHPLC-QE-MS) analysis of YLZ and its drug-containing serum

The quality standards and stability of YLZ were detected by UHPLC-QE-MS. The composition of YLZ and its drug-containing serum were analyzed as follows: a 3 ml sample of three independently decocted YLZ was thawed on ice. After a 30 s vortex, the dection was centrifuged at 12000 rpm (Relative Centrifugal Force (RCF) = 13,800 (× g), Radius (R) = 8.6 cm) for 15 min at 4℃. 300μL of supernatant was transferred to a fresh tube and 1000μL of extracted solution containing 10 μg/mL of internal standard was added, then the samples were sonicated for 5 min in an ice-water bath. After placing 1 h in -40℃, the samples were centrifuged at 12000 rpm (RCF = 13,800 (× g), R = 8.6 cm)for 15 min at 4℃. The supernatant was carefully filtered through a 0.22 μm microporous membrane, then take 200μL from each sample and pooled as Quality Control (QC) samples. Store at -80℃ until the UHPLC- MS analysis.

Four hundred μL of three independent blank serum and YLZ-containing serum samples were added to 40μL of hydrochloric acid(2 mol/L), then the mixture was vortexed for 1 min and followed by incubated for 15 min at 4℃. The vortex and incubate cycle was repeated for 4 times. Add 1.6 mL acetonitrile, then the mixture was vortexed for 5 min and the samples were centrifuged at 12000 rpm (RCF = 13,800 (× g), R = 8.6 cm) for 5 min at 4℃. 1800μL of supernatant was transferred to a fresh tube and nitrogen-dried. The dried samples were reconstituted in 150μL of 80% methyl alcohol containing 10 μg/mL of the internal standard by vortex for 5 min. The constitution was then centrifuged at 12,000 rpm (RCF = 13,800 (× g), R = 8.6 cm) for 5 min at 4℃, and 120μL of supernatant was transferred to a fresh glass vial for LC/MS analysis.

LC–MS/MS analysis was performed on an UHPLC system (Vanquish, Thermo Fisher Scientific) with a Waters Ultra Performance Liquid Chromatography Ethylene Bridged Hybrid (UPLC BEH) C18 column (1.7 μm 2.1*100 mm). The elution condition of the mobile phase (A: water; B: acetonitrile) was as Table 2. An Orbitrap Exploris 120 mass spectrometer coupled with Xcalibur software was employed to obtain the MS and MS/MS data based on the Ionospheric Dispersion Analysis (IDA) acquisition mode. During each acquisition cycle, the mass range was from 100 to 1500, the top four of every cycle were screened and the corresponding MS/MS data were further acquired. Sheath gas flow rate: 30 Arb, Aux gas flow rate: 10 Arb, Ion Transfer Tube Temp: 350 ℃, Vaporizer Temp: 350 ℃, Full ms resolution: 60,000, MS/MS resolution: 15,000, Collision energy: 16/32/48 in NCE mode, Spray Voltage: 5.5 kV (positive) or -4 kV (negative).

**Table 2:** The elution condition of the mobile phase (A: water; B: acetonitrile)

Time/min	Flow rate(μL/min)	Phase A% (water)	Phase B%(Acetonitrile)
0	40	95	5
3.5	40	85	15
6	40	70	30
6.5	40	70	30
12	40	30	70
12.5	40	30	70
18	40	0	100
22	40	0	100
25	40	0	100
26	40	95	5
30	40	95	5

### Animals and experimental protocol

In vitro experiments, twenty-four 8–9-week-old SPF-grade SD female rats (Beijing WTLH, China, SCXK (Beijing) 2021–0011) were housed at the animal experiments building of Capital Medicine University. The environmental conditions were natural light, temperature of 22 ± 2℃, and humidity of 65 ± 5%. The whole rats had free space with enough water and feed. The animal experimental protocol was approved by the Animal Ethics Committee of Capital Medical University (NO.AEEI-2021–131). After seven days of acclimation culture, rats were divided into four groups: Control group, VCD model group (80 mg/kg), E2 valerate group (0.1008 mg/kg), YLZ medium dose group (10.08 g/kg), All groups except the control group were injected intraperitoneally with VCD for 15 days; the control group was replaced with an equal volume of saline. Meanwhile, the YLZ and E2 groups were gavaged with their corresponding drugs for 6 weeks; the control and VCD groups were gavaged with the same volume of saline.

### Estrous cycle monitoring

Vaginal smears were made at 9 a.m. each day for 15 days in the process of model building. Small cotton swabs were soaked in saline, inserted into the vagina of a rat approximately 5 min, rotated clockwise 6 times, then rolled and smeared on a clean slide. After the slide was naturally air-dried, stained with Swiss Giemsa staining solution for approximately 5–10 min, rinsed with water, and observed under an ordinary optical microscope.

### Hormone detection by ELISA

The serum was determined using ELISA assays for the level of FSH, E_2,_ and AMH, following the instructions of the kit.

### Hematoxylin and eosin (HE) staining

The ovaries were isolated and fixed in 4% polyformaldehyde for 48 h and then were processed for paraffin embedding and sectioning. Serial sections of 4 μm thickness were cut with a Leica RM2016 rotator microtome. The sections were dewaxed with xylene, rehydrated with graded concentrations of ethanol, and then stained with hematoxylin and eosin and observed via light microscopy.

### Tissue immunofluorescence

Put the slices in 3 changes of xylene, for 10 min each, then dehydrate in 3 changes of pure ethanol for 5 min each, and wash in distilled water. After repairing antigen was completed, it is naturally cooled. Put the slides 5 min Phosphate Buffered Saline (PBS) (PH7.4) and shake it decoloring shaker 3 times, each time for 5 min. Adding 3% Bovine albumin (BSA) into the tissue and cover it evenly to block, non-specific binding at room temperature for 30 min. (The primary antibody is blocked with 10% donkey serum from goat, and the primary antibody from other sources is blocked with 3% BSA). Adding primary antibody and secondary antibody. The 4',6-diamidino-2-phenylindole (DAPI) solution was dripped into the tissue and incubated at room temperature for 10 min in the dark. Add autofluorescence quencher B solution for 5 min and rinse with running water for 10 min. The Anti-fluorescence quenching was used to seal slides, and collecting images by Fluorescent Microscopy.

### Primary ovarian GCs culture

Female SD rats (21–25 days old, 50 ± 5 g) were subcutaneously injected with pregnant mare serum gonadotropin (PMSG) 50 IU and were sacrificed 48 h later. Removed ovaries were washed immediately with D-Hanks and placed in DMEM medium. GCs were harvested in the medium by 1 ml needle puncture of ovarian follicles and then purified by filtration with 40 μm disposable cell filter mesh. After the centrifugation at 1000 × g for 5 min, the cells were resuspended in medium and counted in a hemocytometer. The cells were seeded in a T25 cell culture bottle and cultured in DMEM medium supplemented with 10% FBS and 1% green streptomycin mixture at 37 ℃ and 5% CO_2_ for 48 h to allow the cells to attach. Cells of logarithmic growth stage were taken and divided into control group, model group (0.5 mM VCD), estradiol group (0.5 mM VCD + 5% E2 containing serum), YLZ group (0.5 mM VCD + 5% YLZ containing serum), HIF1α inhabition group (0.5 mM VCD + 0.5 μM echinomycin (Echi)), HIF1α inhabition + YLZ group (0.5 mM VCD + 0.5 μM Echi + 5% YLZ containing serum).

### Identification of primary ovarian GCs

Cell immunofluorescence identified the primary ovarian GCs. Cells were inoculated in a 24-well plate with a cell patch, After cells had grown to 60% confluence, slides were washed with PBS, fixed in 4% paraformaldehyde, and then permeabilized with 0.5% TritonX, incubated with FSHR overnight, incubated with IgG the next day, dried and mounted, finally, observed and captured picture under a fluorescence microscope.

### Cell proliferation viability determination

Cell Counting Kit-8 (CCK-8) assay measured the cell proliferation viability. After the cells were successfully attached, E2 and YLZ groups were given drugs respectively for 24 h, 48 h, and 72 h, At the end of theculture, cells of the E2 group and YLZ group in 96-well plates were incubated in 100μL DMEM supplemented with 10 μL CCK-8 reagent for 2.5 h at 37℃ and 5% CO_2_ incubator. The optical density (OD) value of each well was measured at a wavelength of 450 nm by a multiscan spectrum. The cell proliferation viability = (the OD value of the test group well -the mean OD value of the blank group)/(the OD value of the negative control group well-the mean OD value of the blank group). Each group was established in four wells.

### ATP level detection

ATP level detection by the chemiluminescence ATP assay kit, according to the manufacturer’s recommendations.

### Cell energy metabolism determination by Seahorse XFe24

The glycolytic metabolic rate was measured on a seahorse XFe24 energy Analyzer(Seahorse Bioscience, USA)0.5 × 10^4^ cells were seeded in an XFe24 culture microplate(Alicelligent, China), and detection of the Extracellular Acidification Rate (ECAR) and Oxygen Consumption Rate (OCR) value according to the operating instructions.

### RT-qPCR

The mRNA expression of HIF1α, Cx43, LDH, HK1, and PEK were detected by RT-qPCR. Granulosa cells were cultured in 6-well plates, Extraction of total RNA was performed with TRIzol reagent after the end of cell intervention in each group. Then the RNA was further reverse-transcribed into cDNA. The following conditions were used for reverse transcription: 42 ℃ for 15 min, 85 ℃ for 5 s, cooling to 4℃ for 5 min, and refrigeration at -20 ℃. The reverse transcription reaction mixture was added to the tubes used for real-time fluorescence quantitative reaction, and the amplification reaction system was as follows: pre-denaturation at 95 ℃ for 10 min, followed by 40 cycles (95 ℃ for 5 s and 60 ℃ for 60 s). The reaction results were analyzed by the relative fold change using the 2 ^−△△CT^ method. Table 3 shows the primer sequences used for real-time fluorescence quantitative reaction of the genes. GAPDH was used as the internal reference gene.

**Table 3:** Primer seguences

Gene	Forward primer	Reverse primer
Rat Cx43	TCTATGGGTTCAGCTTGAGCG	AGATGGTTTTCTCCGTGGGAC
Rat HIF1α	TCGAAGTAGTGCTGATCCTGC	GAAGGACTTGCTGGCTGATCT
Rat HK1	TGGCCTATTACTTCACCGAGC	CGCATGGCGTACAGATACTTG
Rat LDH	TTCATCCACTGAGCTGTCACG	ATTCACACCACTCCACACAGG
Rat PEK	TCGGATACGGCATTTGGCTT	CGTCTTCCACGGTCACTTCG
Rat GAPDH	TGTTCTAGAGACAGCCGCAT	AAATCCGTTCACACCGACCT

### Western blot analysis

The protein expression levels of HIF1α, Cx43, LDH, HK1, and PEK were detected by Western blot. In brief, RIPA lysate and protease inhibitor were added to the cells of each group, centrifuged at 4℃, 12000 rpm for 15 min, and the supernatant was obtained. The protein concentration was determined according to the instructions of the Bicinchoninic Acid (BCA) protein quantitative kit. The total protein was separated by 10% Sodium dodecyl sulfate–polyacrylamide gel electrophoresis (SDS-PAGE) and transferred to a PVDF membrane on ice. And then, the membrane was blocked with 5% skim milk at room temperature for 2 h. The primary antibody was added and incubated overnight at 4℃ and the horseradish peroxidase (HRP) conjugated goat anti-rabbit IgG antibody for 1 h at room temperature. The membranes were exposured with ECL. The relative optical density was assessed by ImageJ software.

### Statistical analysis

Data of the study were showed as mean ± standard deviation (SD), and data were analyzed by SPSS 19.0. One-way Analysis of Variance (ANOVA) was used to analyze statistical differences between different groups. Multiple comparisons were used in post hoc analysis, Least Significant Difference (LSD) was used for homogeneous variances, and Tamhane T2 was used for heterogeneous variances. *P* < 0.05 was identified as a statistically significant difference.

## Results

### Quality control of YLZ and drug-containing serum by UHPLC-MS

The quality control of drug-containing serum, blank serum, and YLZ decocting solution were monitored by UHPLC-MS. We identified 1702 YLZ ingredients and 80 YLZ components entering blood. We showed the top 10 of it (Table 4, Fig. 1B). The chromatograms in positive and negative ion mode are as Fig. 1A. To further determine the effect of Yu linzhu ingredients on blood, the YLZ components entering blood were associated with the measured serum FSH, AMH, E2 levels of Rats (Fig. 1C).

**Table 4:** YLZ ingredients

English Name	Class	InChikey	Formula
Epigallocatechin	Polyphenols	XMOCLSLCDHWDHP-IUODEOHRSA-N	C15H14O7
Myricetin	flavonoids	IKMDFBPHZNJCSN-UHFFFAOYSA-N	C15H10O8
Amaranthussaponin II	Isopentenol fats	MMFXDLCKZVIKRE-UHFFFAOYSA-N	C48H74O20
Bixin	Carotenoids	RAFGELQLHMBRHD-IFNPSABLSA-N	C25H30O4
geniposidic acid	Iridoid glucoside	ZJDOESGVOWAULF-OGJQONSISA-N	C16H22O10
Myricolal	terpenoids	IFVLEXPVJXHCAY-UHFFFAOYSA-N	C30H48O2
Santene	/	LSIXBBPOJBJQHN-UHFFFAOYSA-N	C9H14
Trimethyloxazole	oxazole	ZRLDBDZSLLGDOX-UHFFFAOYSA-N	C6H9NO
Tacrolimus	Terpenoids	QJJXYPPXXYFBGM-LFZNUXCKSA-N	C44H69NO12
Apigenin 7-O-glucuronide	Flavonoids	JBFOLLJCGUCDQP-ZFORQUDYSA-N	C21H18O11

### The estrous cycle of POI rats

**Fig. 2:**
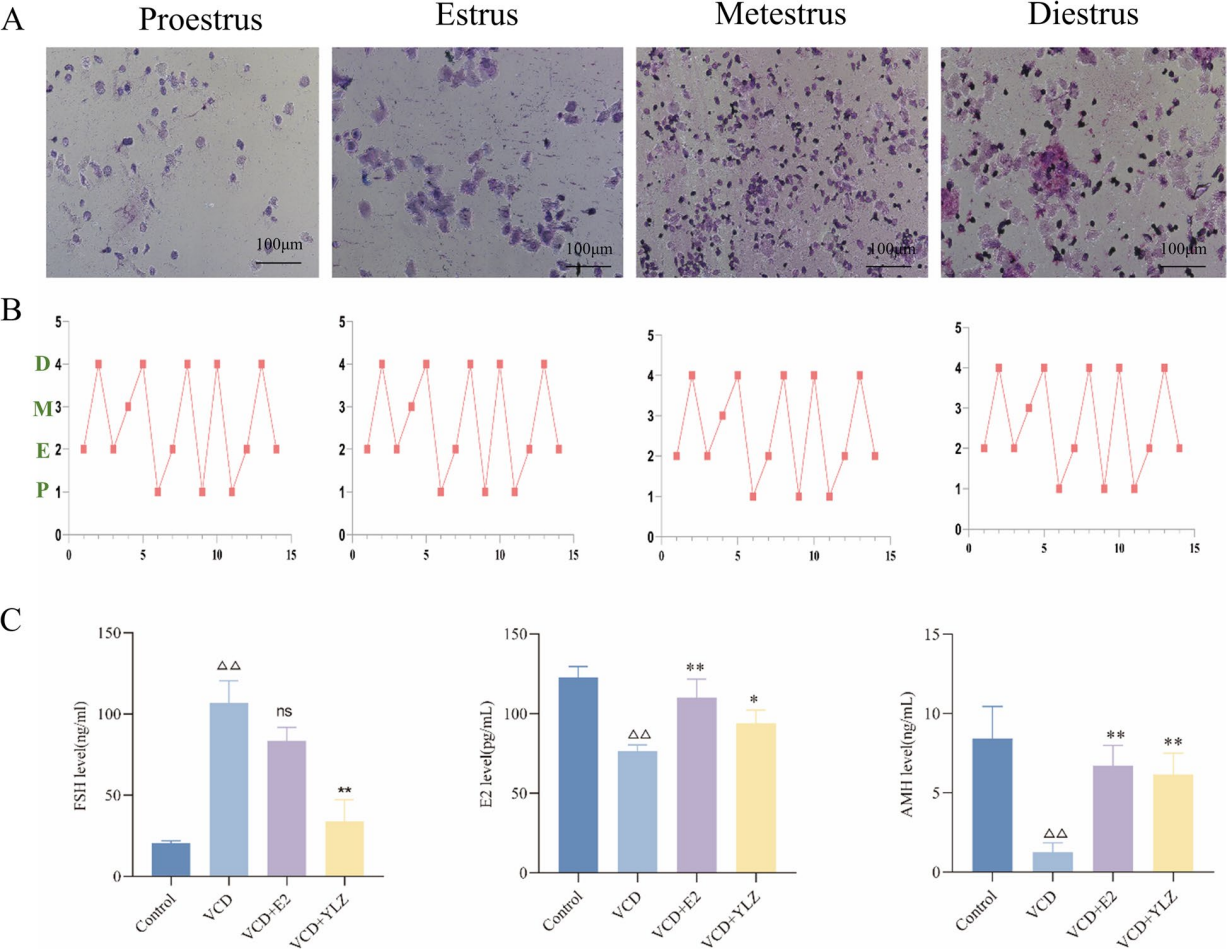
The estrous cycle of VCD model rats and hormone levels of each group. **A** Estrous cycles disorder within 15 days of intraperitoneal injection with VCD. **B** The dynamic changes in the estrus cycle in 15 days (P = proestrus; E = estrus; M = metartus; D = diestrus). **C** The hormone levels in each group. ^△△^ *P* < 0.01 versus control group; ^*^ *P* < 0.05, ^**^ *P* < 0.01 versus VCD group

The estrous cycle of rats in the process of establishing the POI model using VCD is shown in (Fig. 2AB). There was majority of nucleated epithelial cells in proestrus, the keratinized epithelial cells in estrus, the white blood cells in metestrus, and keratinized epithelial cells, nucleated epithelial cells and white blood cells had no significant differences in the proportion in diestrus. A significant disorder of the rats were observed during the process of establishing the POI model, which was manifested by lengthening or shortening of the estrous cycle.

### YLZ regulated the hormone levels in POI rats

The serum levels of the rats' hormones were measured by ELISA (Fig. 2C). The rats in the VCD group had higher FSH levels than those in the other groups, and the AMH and E2 levels were lower than others (*P* < 0.01).

### YLZ improved the ovarian tissue pathology

**Fig. 3:**
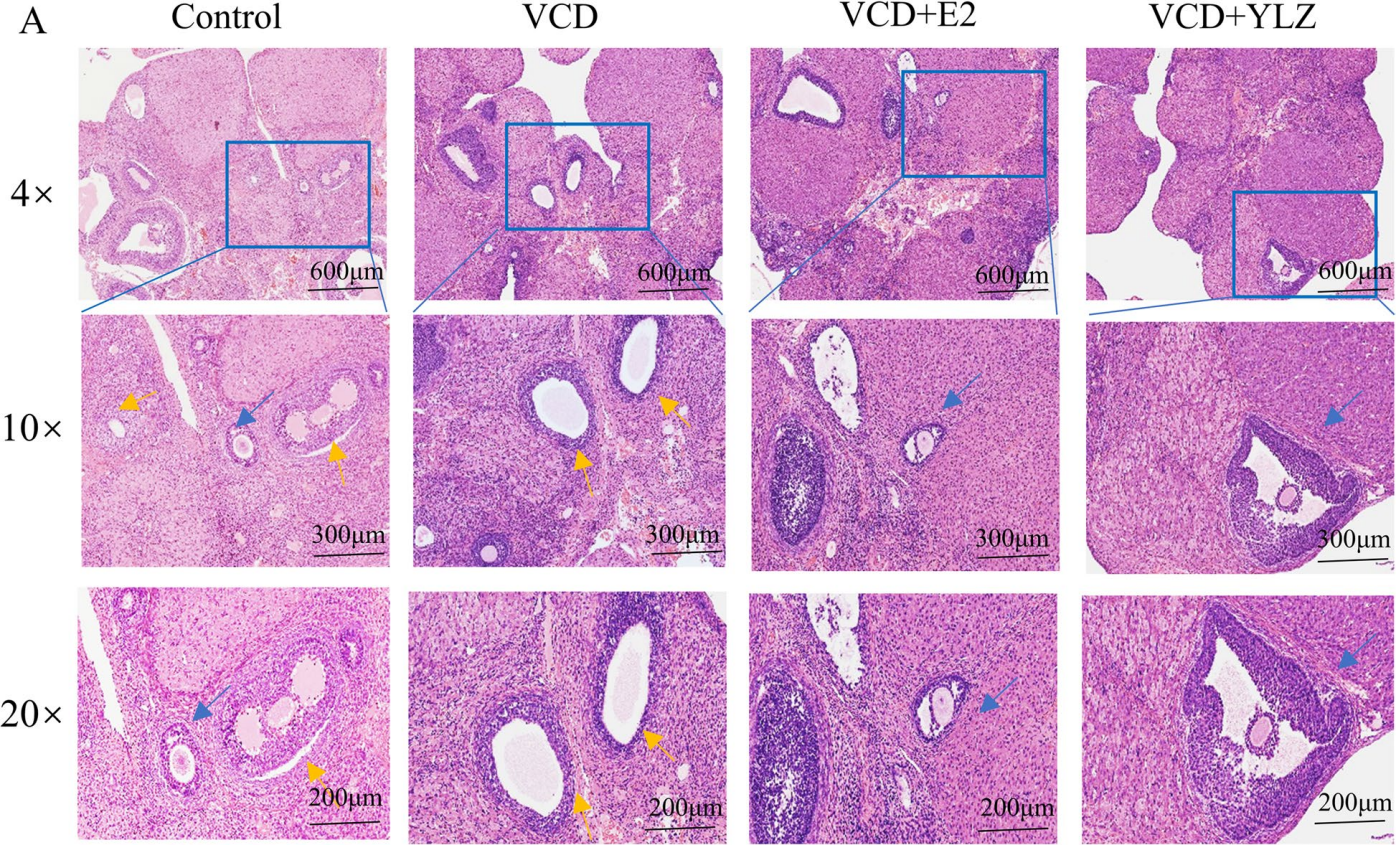
Ovarian tissue pathology of each group. Blue arrows are normal follicles, yellow arrows are atretic follicles

The ovarian tissue morphology was observed under a microscope after hematoxylin and eosin (H&E) staining. The follicular quantity of the control group was more numerous than the VCD group, and GCs arranged neatly, oocyte morphology was regular, and the number of atretic follicles was less than that of the VCD group (Fig. 3). The number of the atretic follicular were dramatically increased in VCD group. On the contrary, the GCs layer was more arranged, oocyte morphology recovered regularly, and the number of normal follicular increased in VCD + E2 and VCD + YLZ groups.

### YLZ increased the expression of CX43 in ovarian tissues

**Fig. 4:**
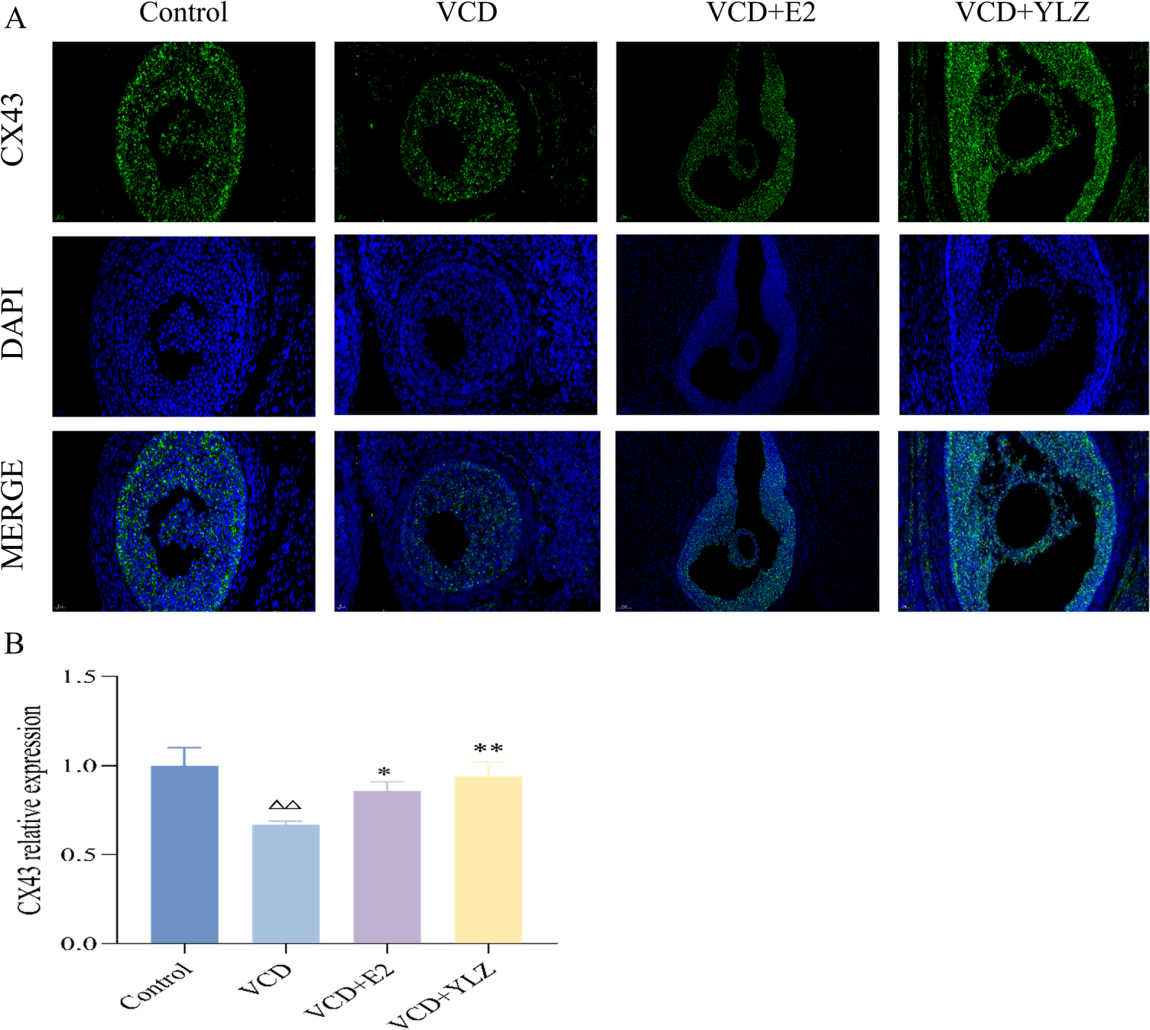
The location and expression of CX43 in ovarian tissues. **A** The immunofluorescence of CX43 in each group (40 × , scale label = 20 μm). **B** The CX43 relative expression in each group. ^△△^ *P* < 0.01 versus control group; ^*^ *P* < 0.05, ^**^ *P* < 0.01 versus VCD group

We used tissue immunofluorescence to identify the location and expression of CX43 in ovarian tissues (Fig. 4A). We found that compared with the Control group, the cumulus GCs that wrapping oocyte were absent and the CX43 protein secreted by them were decreased obviously in the follicle of VCD group (*P* < 0.01), while the oocytes in each administration group were found to be intact and regular, with cumulus GCs outside, and the expression of CX43 protein was significantly improved compared with that in the VCD group (*P* < 0.05, *P* < 0.01).

### Identification of primary ovarian GCs

**Fig. 5:**
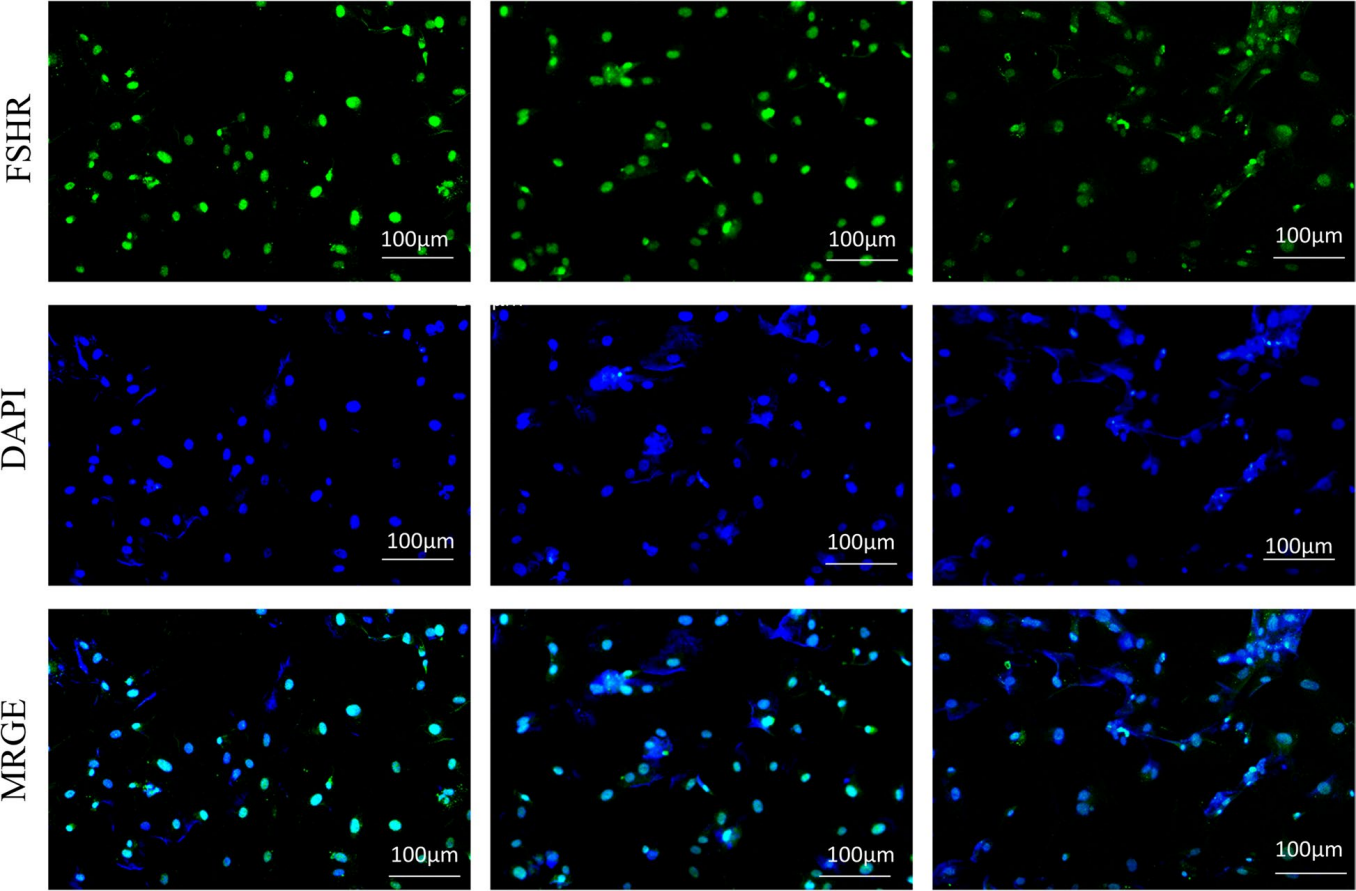
Identification of primary ovarian GCs under the fluorescence microscope (10 ×)

We used cellular immunofluorescence to identify primary ovarian GCs. FSHR, a specific antibody of ovarian GCs, was used for the identification of primary cell purity, we stained them and acquired fluorescence images with a fluorescence microscope, calculated the percentage of blue and green fluorescence coincidence finally. The visual fields of 3 holes were randomly selected to calculate the purity (purity = number of positive cells/total number of cells × 100%). The experimental results showed that the purity of primary ovarian GCs was greater than 98%, therefore subsequent experiments could be conducted (Fig. 5).

### YLZ improved the proliferation and ATP of ovarian GCs

**Fig. 6:**
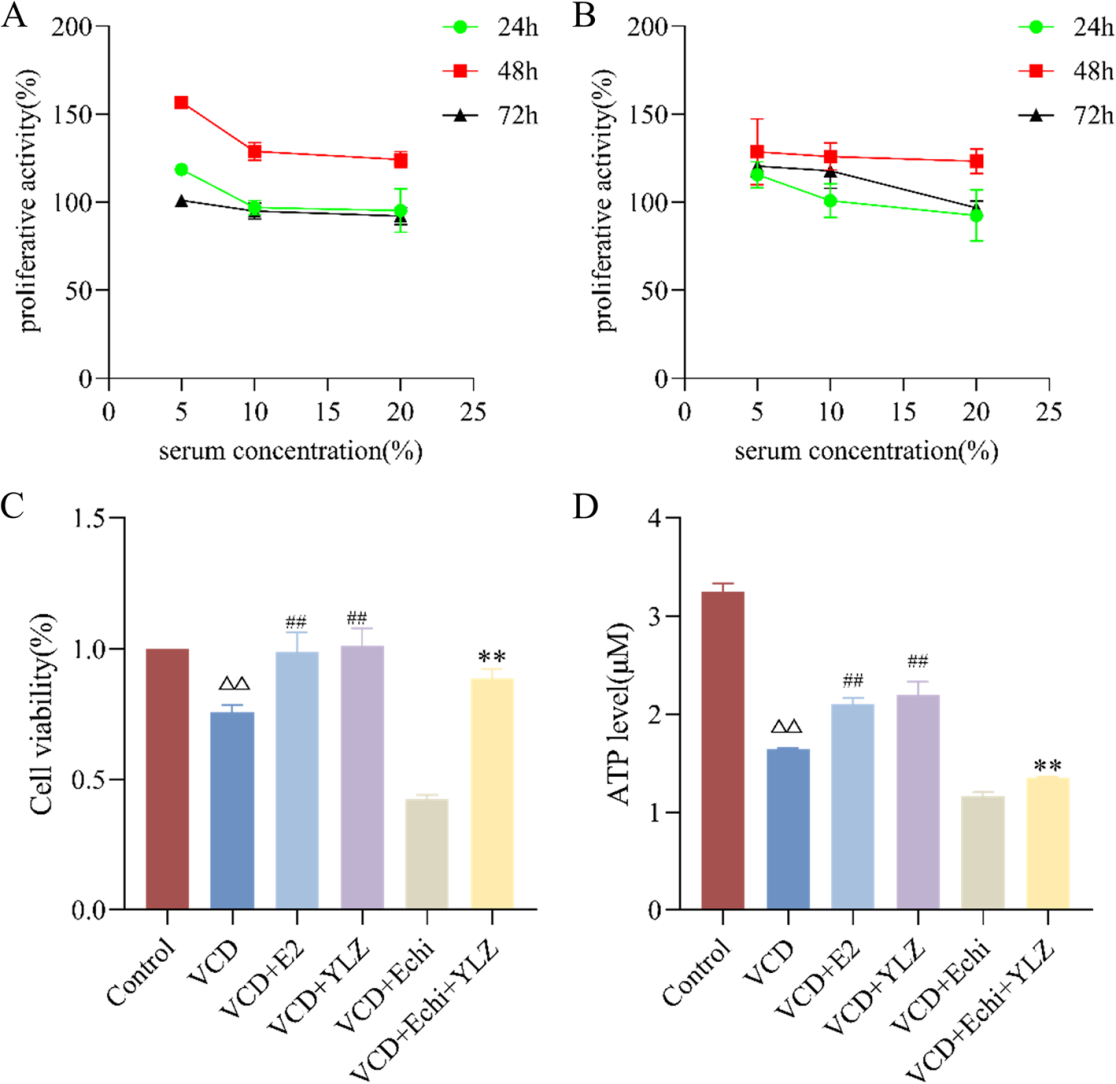
The proliferation and ATP of ovarian GCs. **A** Effect of YLZ at different times and concentrations on ovarian GCs. **B** Effect of E2 at different times and concentrations on ovarian GCs. **C** Cell proliferation viability of E2 and YLZ at 48 h, 5% concentration on VCD and Echi-induced ovarian granulosa cells of each group.^△△^ *P* < 0.01 versus control group; ^##^ *P* < 0.01 versus VCD group; ^**^ *P* < 0.01 versus VCD + Echi group

We used the CCK8 assay to confirm the time and concentration of E2 and YLZ administration. We found that with the increase of dose concentration, cell viability decreased, and the 48 h and 5% concentrations were the optimal conditions to conduct the subsequent experiments (Fig. 6AB). We set up 6 groups and the study showed that compared with the control group, cell viability decreased by nearly 25% in the VCD group (*P* < 0.01). However, when VCD was co-applied with YLZ and E2, we found that YLZ and E2 could rescue the VCD-induced cell damage (*P* < 0.01). Compared with the VCD + Echi group, cell viability was increased by approximately 40% in the VCD + Echi + YLZ group (*P* < 0.01), this result indicated that the protective effect of YLZ on damaged GCs (Fig. 6C). And ATP test kit was used to detect the ATP of each group. We found similar results with proliferation from the CCK8 assay (Fig. 6D).

### YLZ increased the glycolytic metabolism level of ovarian GCs

**Fig. 7:**
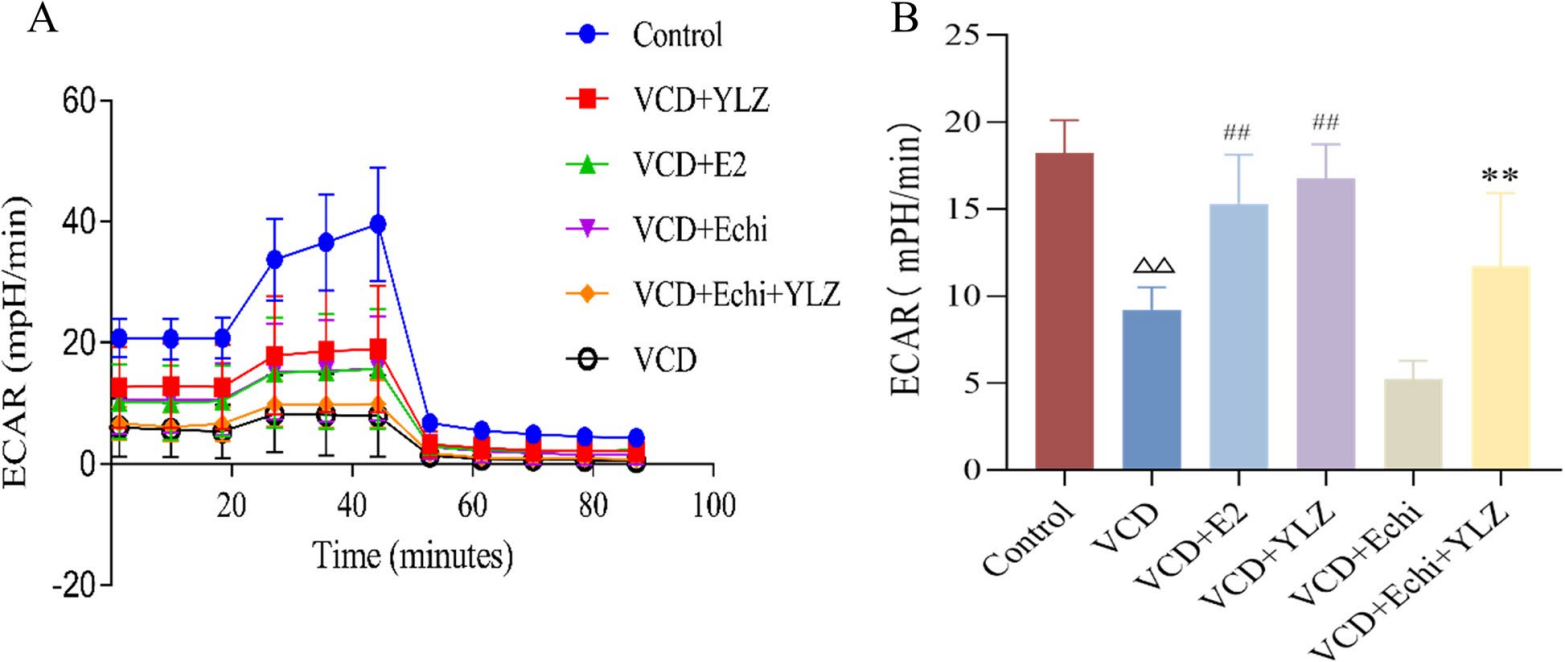
The glycolytic metabolism level of ovarian GCs. **A** Glycolysis rate diagram of each group. **B** ECAR levels of each group. ^△△^ *P* < 0.01 versus control group; ^##^ *P* < 0.01 versus VCD group; ^**^ *P* < 0.01 versus VCD + Echi group

We used seahorse XFe24 to detect the glycolytic metabolism level of cells. With the extension of time, the glycolysis rate of each group decreased continuously (Fig. 7A). Compared with the control group, EACR levels in the VCD group decreased nearly half of the control group (*P* < 0.01). Nevertheless, E2 and YLZ reversed this kind of damage (*P* < 0.01). What’s more, in comparison to the VCD + Echi group, the metabolism level of the VCD + Echi + YLZ group also increased (*P* < 0.01) (Fig. 7B).

### YLZ increased the expression of energy metabolism-related mRNA and proteins in ovarian GCs

**Fig. 8:**
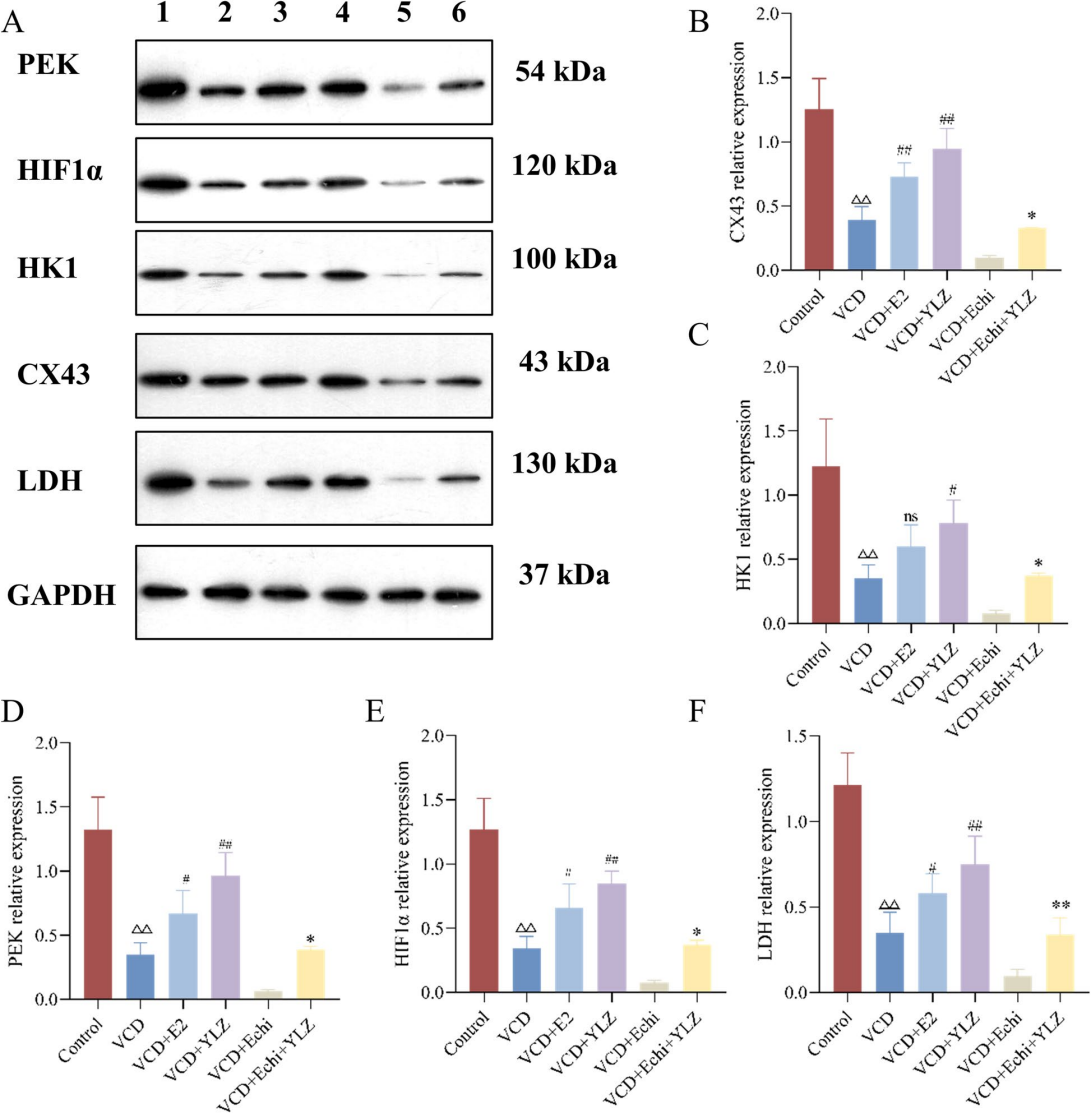
YLZ increased the protein expression of the HIF1α /CX43 pathway in ovarian GCs. **A** Western blot analysis of HIF1α, CX43, PEK, HK1, and LDH protein levels in ovarian GCs, GAPDH was used as an internal control. **B-F** Statistical analysis of HIF1α, CX43, PEK, HK1, and LDH relative protein expression. ^△△^ *P* < 0.01 versus control group; ^#^ *P* < 0.05 and ^##^ *P* < 0.01 versus VCD group; ^*^ *P* < 0.05 and ^**^ *P* < 0.01 versus VCD + Echi group

**Fig. 9:**
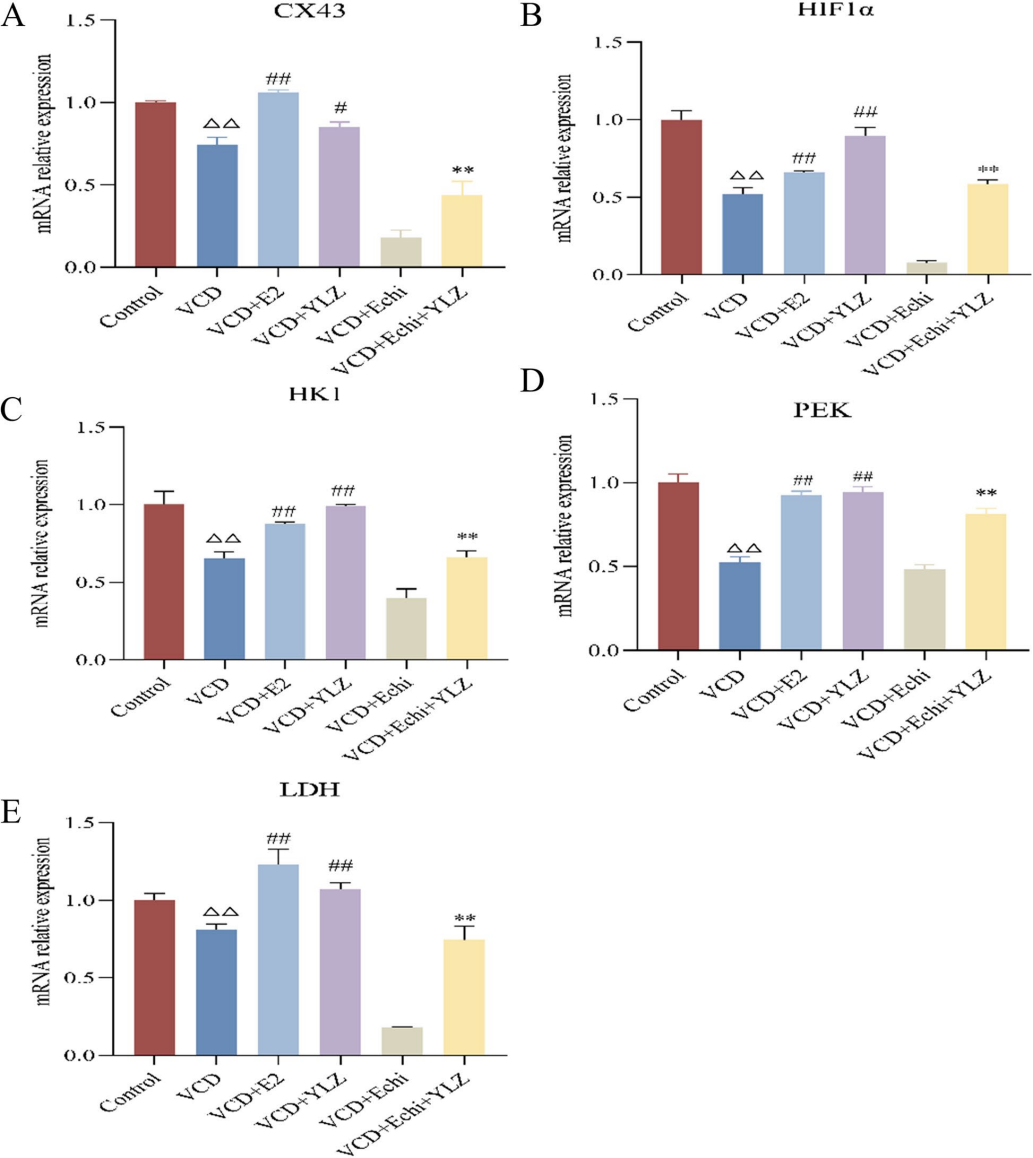
YLZ increased the mRNA expression of the HIF1α /CX43 pathway in ovarian GCs. (A-E) mRNA analysis of HIF1α,CX43, PEK, HK1,and LDH relative levels in ovarian GCs.^△△^ *P* < 0.01 versus control group; ^#^ *P* < 0.05 and ^##^ *P* < 0.01 versus VCD group; ^**^ *P* < 0.01 versus VCD + Echi group

RT-qPCR and the western blot were applied to detect the further mechanism by which YLZ improves the proliferation and energy metabolism of ovarian GCs (Fig. 8), Which showed that a decrease of Cx43, HIF1α, LDH, HK1, PEK mRNA, and protein expression in VCD group, compared with the control group (*P* < 0.05 or *P* < 0.01). By contrast, E2 and YLZ completely reversed the decrease mentioned above (*P* < 0.05 or *P* < 0.01). In the VCD + Echi + YLZ group, YLZ further alleviated the decline of related genes or proteins inhibited by HIF1α protein (*P* < 0.05 or *P* < 0.01). The results above imply that the HIFα /Cx43 signaling pathway may be the mechanism of energy metabolism in ovarian GCs of which YLZ acts (Fig. 9).

## Discussion

In this study, we found that YLZ improved ovarian function by alleviating hormone level, ovarian morphology, follicular development, the proliferation and energy metabolism of GCs, as well as expression of some energy metabolism-related proteins and genes both in vivo and in vitro.

Firstly, we used UHPLC-QE-MS to conduct quality control and stability of YLZ. The chromatographic results showed that there was no significant difference in the detected drug components under the positive and negative ion modes, indicating the stability of the drug quality of YLZ. We intermingled the detected YLZ with the drug-containing serum and found that as many as 80 Chinese medicine components successfully entered the blood and participated in the biological process in the body. Meanwhile, the heat map of hormones and the drug-containing serum also indicated that YLZ regulated the serum hormone level in rats through a multi-component pathway, thus playing a role in the treatment of POI.

There was study has shown that the estrous cycle and serum hormone levels of animals were disordered after the establishment of the POI model [23]. The normal estrous cycle includes four stages: proestrus, estrus, metestrus, and diestrus, which are affected and regulated by gonadal hormones [24]. After the decline of ovarian function, the body is in a state of low estrogen and high gonadotropin caused by hypogonadism, which is manifested by the increase of serum FSH level and the decrease of serum E2 level, and the disturbance of the estrous cycle of animals [25]. As the most reliable and specific biochemical index to measure ovarian reserve function, AMH level can directly and accurately predict the number of reserve follicles in the ovary, and the decrease in AMH level indicates the decline of ovarian function [23, 26]. In this study, it was confirmed that YLZ can distinctly improve the hormonal disorder induced by VCD, thereby improving ovarian function.

Antral follicles are the most important factor in measuring functional ovarian reserve, which is closely related to serum AMH level and ovarian follicle number [27]. Therefore, we carried out a pathological section staining analysis on the ovaries of each group. H&E staining results showed that the ovarian function decreased after the application of the VCD model, the primordial follicles decreased, the oocyte morphology became irregular, the GCs arrangement was disorderly, and the atretic follicles increased. Combined with the results YLZ can increase the number of antral follicles and improve follicle development state.

The follicle is the functional unit of the ovary, and its development involves complex interactions between multiple cell types, among which the intercellular communication between oocytes and GCs is a vital driving force for follicle development [28] GCs are the largest cell group in the follicle microenvironment, and their healthy proliferation and differentiation are crucial for follicles development [29]. The energy metabolism of GCs is a key factor affecting their proliferation, Which is more inclined to produce ATP by glycolysis for energy metabolism compared with most cells that rely on oxidative phosphorylation as the most important way of energy metabolism [30]. In this study, we have found that YLZ can significantly increase the glycolytic metabolism of GCs, produce ATP, and increase the proliferation of GCs through CCK8 and XFe24 glycolytic metabolic rate measurement experiments. HIF1α is the most direct target protein that regulates glycolytic metabolism, it is the regulatory subunit of HIF1, also known as the functional subunit, and plays a key role in the transcriptional activity of HIF1, it is mainly expressed in the nucleus, and its content is regulated by external oxygen concentration [31, 32]. Cx43 is a specific protein secreted by GCs, mediating the gap junction within GCs to provide the transfer of glycolytic energy metabolites, promoting the proliferation of GCs and the formation of antral follicles [28, 33]. We speculated that YLZ may affect the glycolysis of GCs through the HIF1α/Cx43 pathway. To further confirm the hypothesized results, we adopted real-time fluorescent quantitative PCR and western blot to detect the expressions of HIF1α, CX43, and related glycolysis genes and proteins. Echinomycin has been widely used as an effective inhibitor of HIF1α, it was further selected as a HIF1α inhibitor to determine the molecular mechanism by which YLZ acted on POI [34, 35]. In this research, we found that YLZ can ameliorate ovarian injury and hormone disorder which is VCD-induced, promotes GCs proliferation and energy metabolism by regulating levels of HIF1α, CX43, and related glycolytic genes and proteins.

## Conclusion

In conclusion, our results showed the protective effect of YLZ on ovarian function and the positive influences on GCs proliferation and energy metabolism. YLZ is expected to improve POI status and even restore ovarian function in rats, increase glycolytic metabolism of GCs through the HIF1α/Cx43 pathway, promote proliferation, and improve follicle development. As a traditional Chinese medicine formula, the multiple targets and pathways of YLZ urgently and deeply deserve to be further explored. However, the potential adverse effects of YLZ will require further study.

### Supplementary Information

**Supplementary Material 1.**

**Supplementary Material 2.**

**Supplementary Material 3.**

**Supplementary Material 4.**

## Data Availability

No datasets were generated or analysed during the current study.
