# Supplementary figures and images for: Yu Linzhu alleviates primary ovarian insufficiency in a rat model by improving proliferation and energy metabolism of granulosa cells through hif1α/cx43 pathway
Source: J Ovarian Res. 2024 Apr 26;17:89. doi: 10.1186/s13048-024-01408-1 (PMC11046760; doi:10.1186/s13048-024-01408-1)

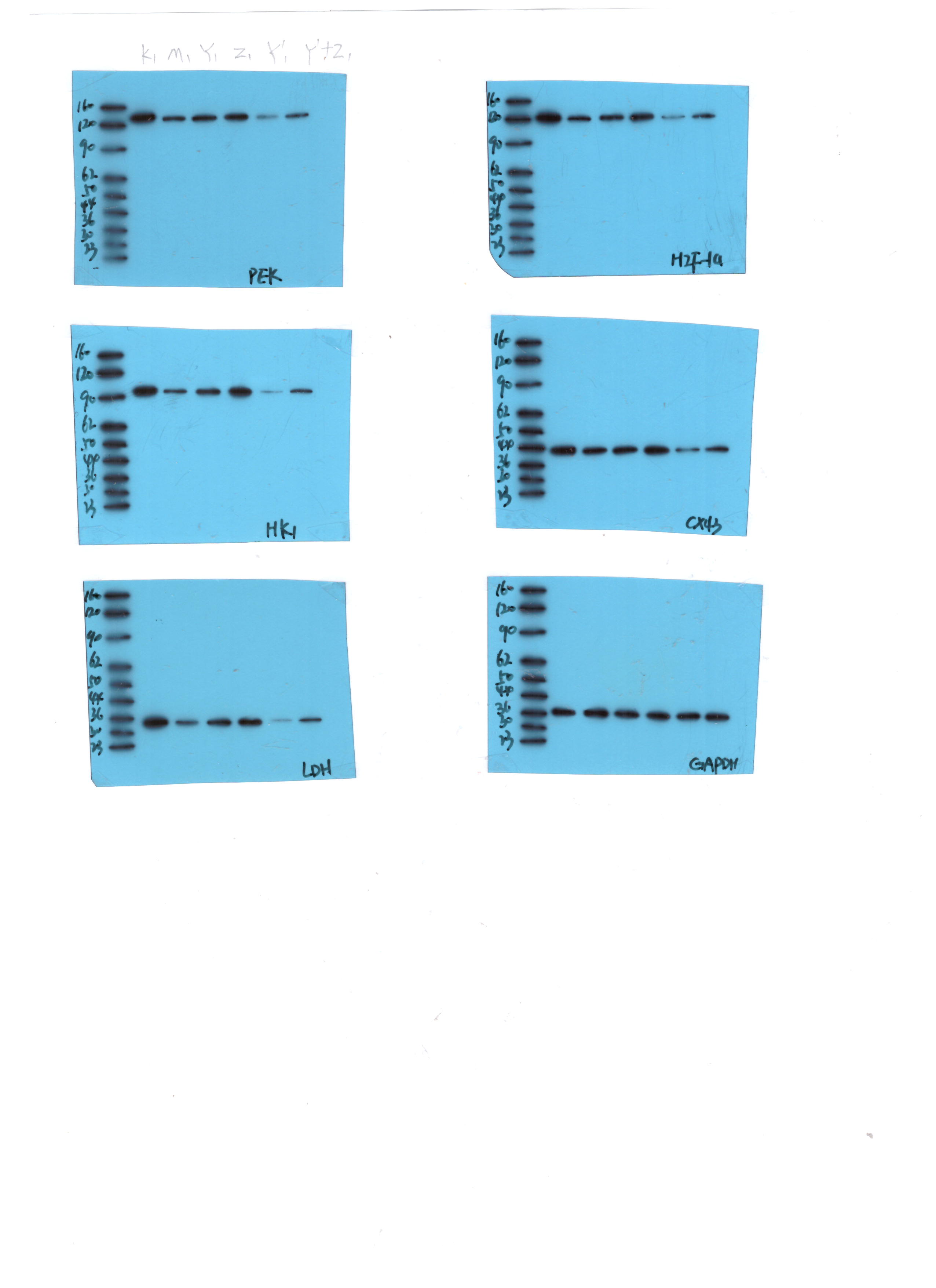

Supplement: Supplementary file 2 — Supplementary Material 2. [file 13048_2024_1408_MOESM2_ESM.tif]

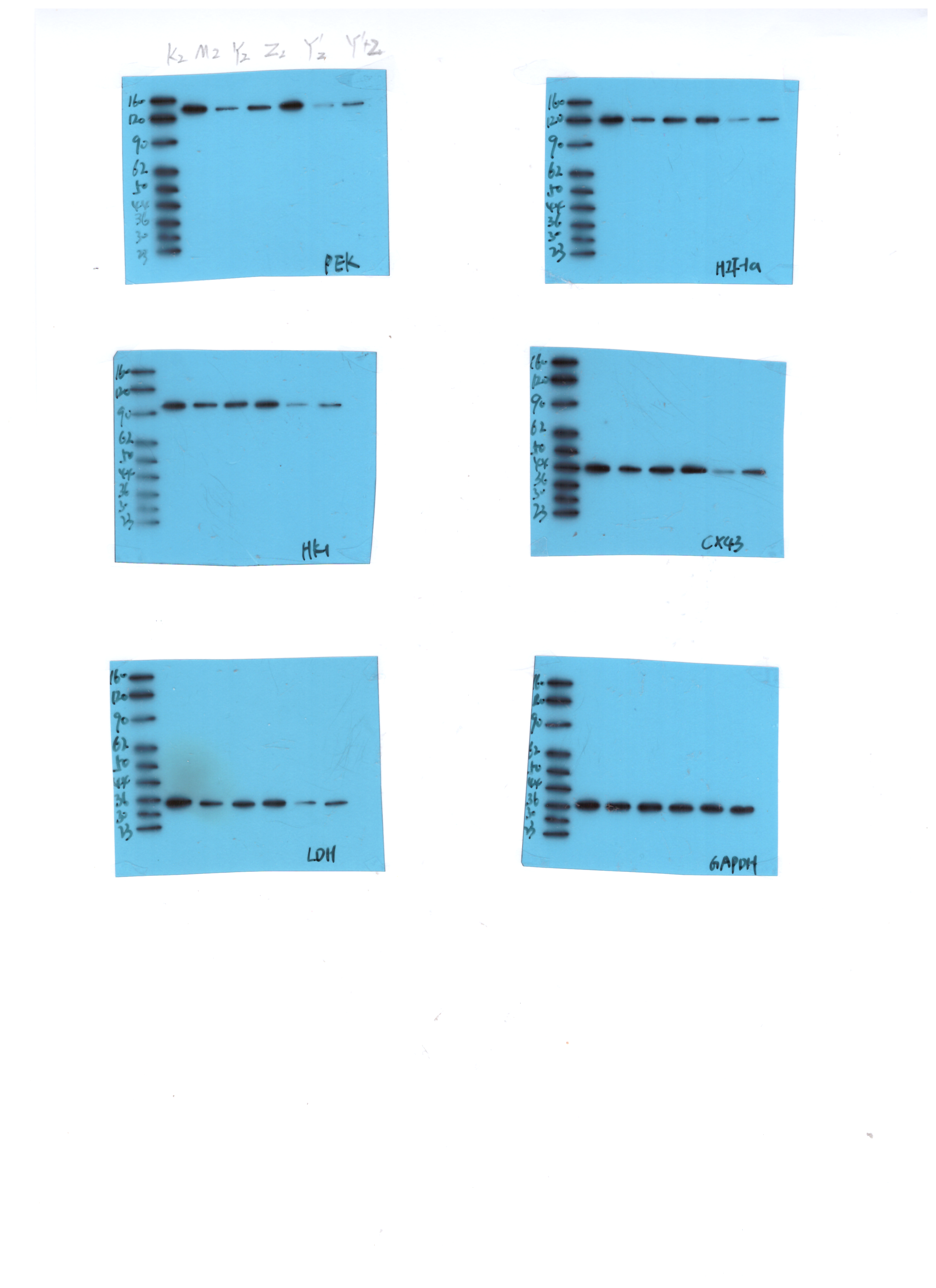

Supplement: Supplementary file 3 — Supplementary Material 3. [file 13048_2024_1408_MOESM3_ESM.tif]

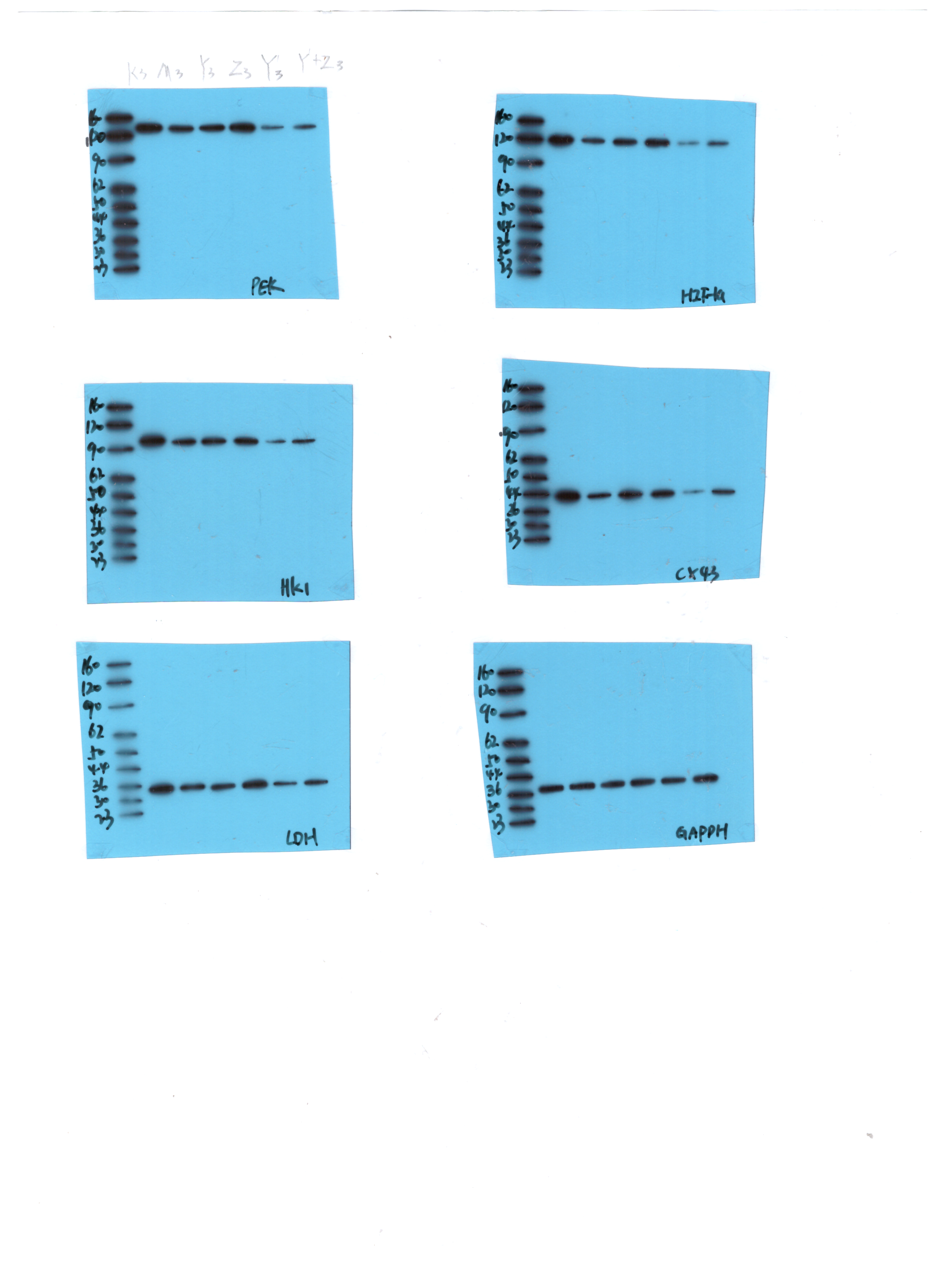

Supplement: Supplementary file 4 — Supplementary Material 4. [file 13048_2024_1408_MOESM4_ESM.tif]
